# P-1047. The Epidemiology of Sporotrichosis: Clinical Features, Risks Factors and Associated Mortality, a Global Research Network Analysis

**DOI:** 10.1093/ofid/ofae631.1237

**Published:** 2025-01-29

**Authors:** Samantha Schapiro, Nicholas Pulciano, Julian Galindo, Andrés F Henao Martínez, George R Thompson, Daniel B Chastain

**Affiliations:** Saint Joseph Hospital Internal Medicine Residency, Wheat Ridge, Colorado; Rocky Vista University College of Osteopathic Medicine, COLORADO SPRINGS, Colorado; Universidad Libre, bogota, Distrito Capital de Bogota, Colombia; University of Colorado Anschutz Medical Campus, Aurora, Colorado; University of California Davis Medical Center, Sacramento, CA; University of Georgia College of Pharmacy, Albany, GA

## Abstract

**Background:**

Sporotrichosis is a fungal infection affecting thousands in South America, yet is uncommon globally, where it is not a reportable disease. This leads to a limited understanding of its epidemiology and clinical features.

The study aimed to identify and characterize the clinical features, geographic distribution, risk factors, and outcomes of sporotrichosis across a multicenter United States (US)-based network to improve diagnostic and treatment strategies.

**Figure:**
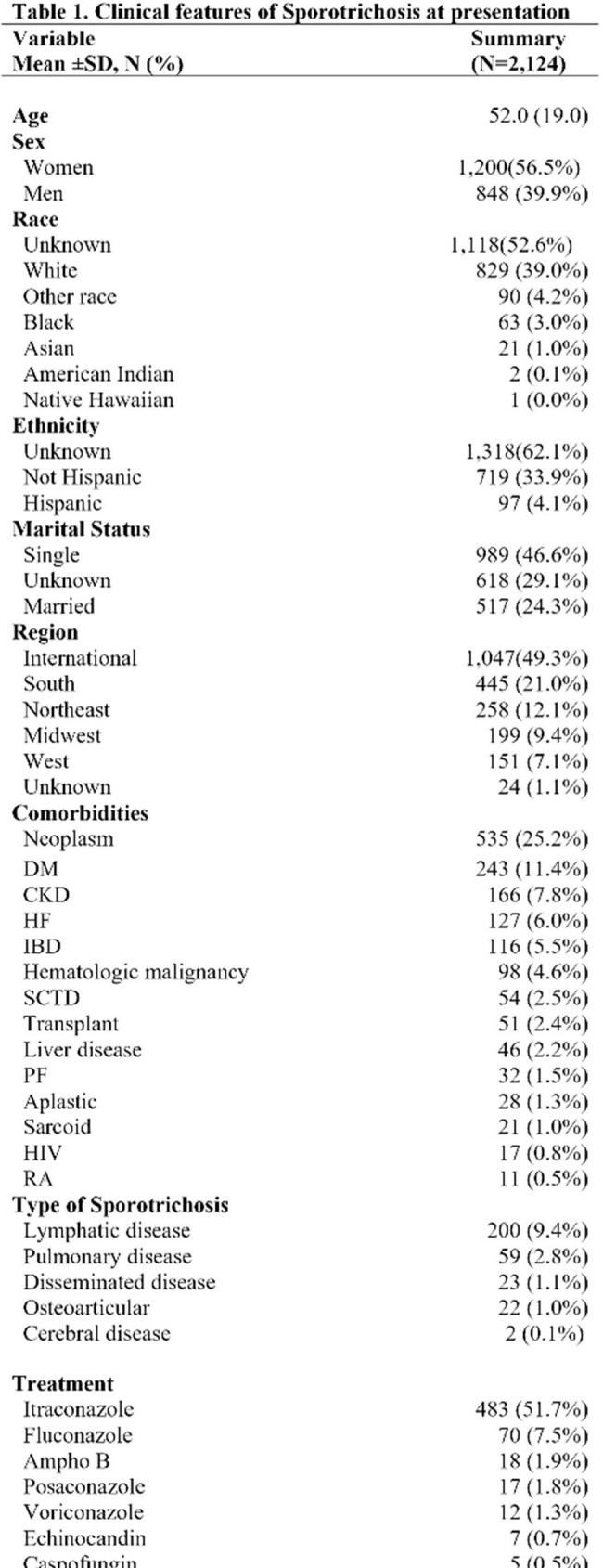

**Methods:**

The study used the TriNetX global research network database to identify patients with sporotrichosis defined by ICD-10-CM code B42. A manual chart review was conducted to verify the reliability of the code in 60 patients. Data collected included demographics, comorbidities, lab results, and outcomes. The primary outcome was mortality and hospitalization. Bivariate analysis was performed to compare survivors with non-survivors in one year.

**Figure:**
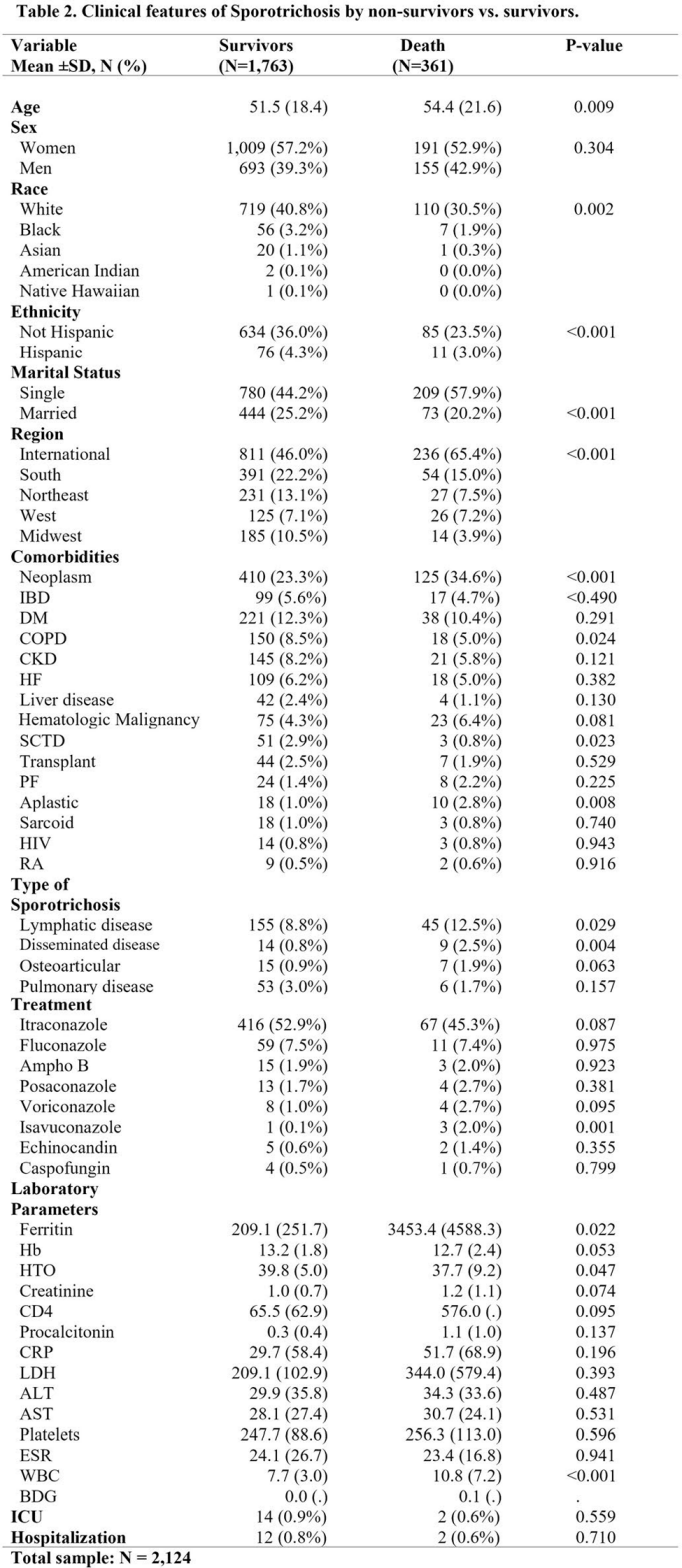

**Results:**

The study included 2,124 patients with sporotrichosis, with a manual chart diagnosis validation rate of 95%. The average age was 52, with 56% women and 40% Caucasian. Common comorbidities were neoplasms, diabetes mellitus (DM), and chronic obstructive pulmonary disease (COPD). Most cases were unspecified, but among those with a specified subtype, lymphatic was the most common (9.4%), followed by disseminated (1.1%). Itraconazole was the most common first-line treatment. The one-year mortality was 17% (Table 1). The Southeast had the most frequent cases in the US (Figure 1). Age, unmarried marital status, and location in south or international regions were significantly associated with mortality. Higher ferritin levels, lower hematocrit, and higher leukocyte count also indicated increased mortality (Table 2). There were 17 patients with both sporotrichosis and HIV; 82% were men with an average age of 56. The average CD4 count was 65.5, with a one-year mortality of 17.6% (Table 3).

**Figure:**
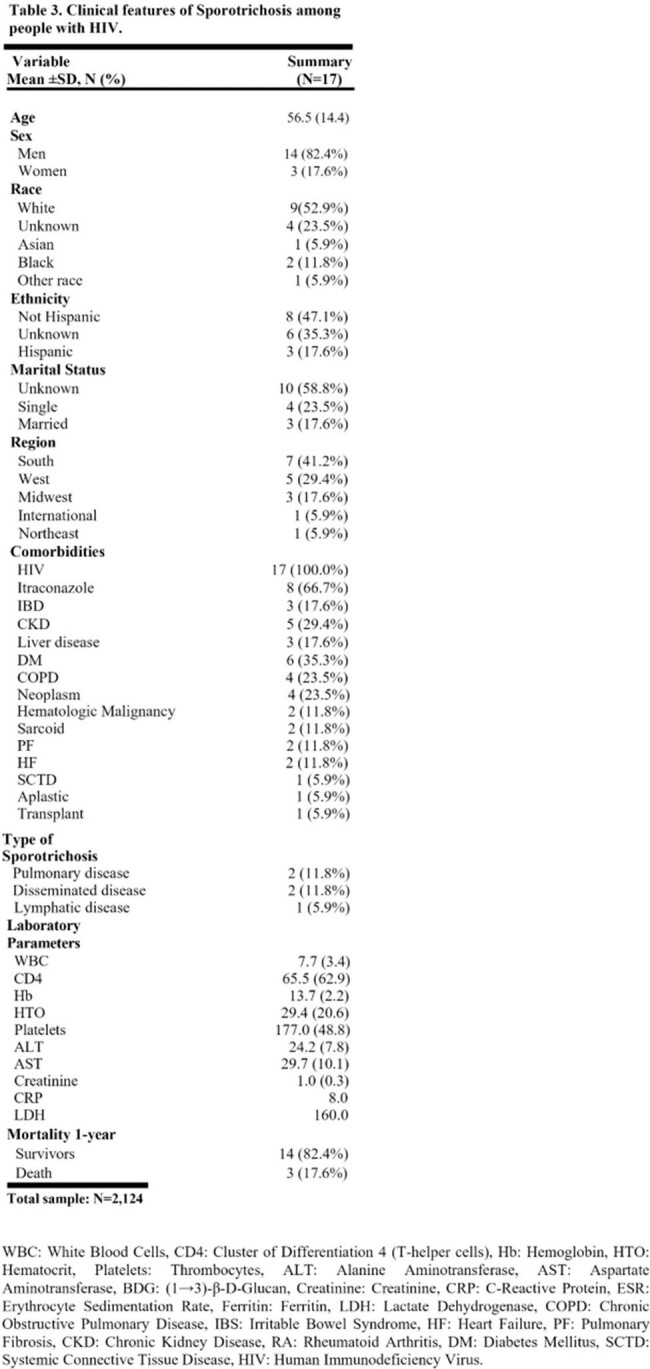

**Conclusion:**

Patients with sporotrichosis had low rates of extracutaneous disease. Age, unmarried marital status, geographic location, and specific comorbidities such as neoplasms, DM, and COPD are significant factors affecting sporotrichosis survival. Despite their compromised immune status, patients with HIV did not have worse outcomes than the general sporotrichosis population.

**Figure:**
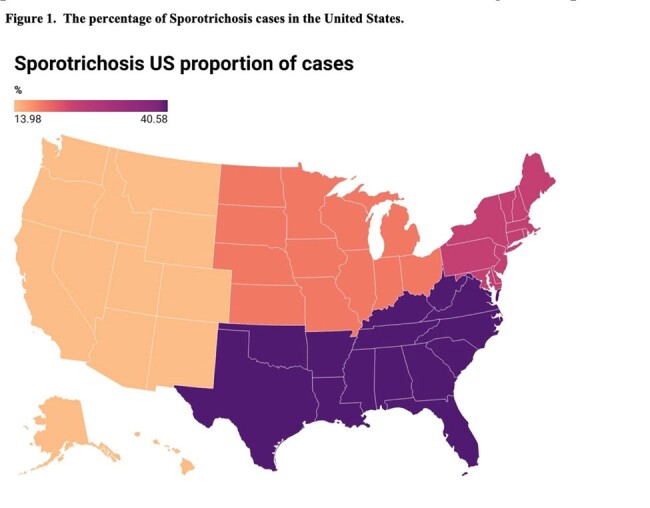

**Disclosures:**

**George R. Thompson, III, MD**, Astellas: Advisor/Consultant|Cidara: Advisor/Consultant|Cidara: Grant/Research Support|F2G: Advisor/Consultant|F2G: Grant/Research Support|Melinta: Advisor/Consultant|Melinta: Grant/Research Support|Mundipharma: Advisor/Consultant|Mundipharma: Grant/Research Support|Pfizer: Advisor/Consultant

